# *Neospora caninum* infection during early pregnancy in cattle: how the isolate influences infection dynamics, clinical outcome and peripheral and local immune responses

**DOI:** 10.1186/1297-9716-45-10

**Published:** 2014-01-30

**Authors:** Javier Regidor-Cerrillo, David Arranz-Solís, Julio Benavides, Mercedes Gómez-Bautista, José Antonio Castro-Hermida, Mercedes Mezo, Valentín Pérez, Luis Miguel Ortega-Mora, Marta González-Warleta

**Affiliations:** 1 Animal Health Department, SALUVET, Faculty of Veterinary Sciences, Complutense University of Madrid, Ciudad Universitaria s/n, 28040 Madrid, Spain; 2 Livestock Health and Production Institute (ULE-CSIC), Grulleros, 24346 León, Spain; 3 Laboratory of Parasitology, Agricultural Research Centre of Mabegondo (CIAM-INGACAL), Xunta de Galicia, Carretera AC-542 de Betanzos a Mesón do Vento, km 7, 15318 Abegondo, A Coruña, Spain

## Abstract

This work studies the influence of *Neospora caninum* intra-species diversity on abortion outcome, infection dynamics in terms of parasite dissemination and peripheral-local immune responses in pregnant cattle. Animals were intravenously inoculated at day 70 of pregnancy with 10^7^ tachyzoites of two isolates showing marked differences in virulence in vitro and in pregnant mouse models: Nc-Spain7, a high virulence isolate, and Nc-Spain8, a low-to-moderate virulence isolate. After inoculation, pregnancy was monitored, and dams were culled when foetal death was detected. Foetal mortality occurred in all infected heifers between days 24 and 49 post-infection (pi), however, it was detected sooner in Nc-Spain7-infected animals (median day = 34) than those inoculated with Nc-Spain8 (median day = 41) with a trend towards significance (*P* < 0.11). Similar histological lesions were observed in placentomes and in most of the foetuses from the two infected groups. However, parasites were more frequently detected in the placenta and foetuses by PCR and in the foetal brain by immunohistochemistry in Nc-Spain7-infected animals. Specific antibodies were detected starting at day 13 post-infection in all infected cattle, with higher IgG levels in Nc-Spain7-infected group. IFN-γ and IL-4 profiles also varied between infected groups in PBMC stimulation assays. Infected animals showed significant increases in their cytokine mRNA levels (IFN-γ, IL-4, IL-10, IL-12p40 and TNF-α) in the caruncle at time of foetal death. Differences between the infected groups were also observed for cytokine profiles. These results demonstrate the influence of the *N. caninum* isolate on foetal death outcome, infection dynamics and immune responses in cattle.

## Introduction

*Neospora caninum* is an obligate intracellular apicomplexan parasite with a complex heteroxenous life cycle in which the domestic dog and other canids act as definitive hosts and different ungulates, including cattle, act as natural intermediate hosts [1-3]. Cattle can become infected via the ingestion of oocysts (horizontal transmission) and transplacentally as a result of this primary infection by oocysts (exogenous transplacental transmission) or by recrudescence of a chronic infection (endogenous transplacental transmission) of the dam during pregnancy; each route has distinct pathogenic, immunological and epidemiological consequences [4,5]. *N. caninum* is transmitted transplacentally in cattle very efficiently. Infection by exogenous or endogenous transmission in pregnant cows can induce damage to the foetus in the uterus and abortion or produce a still-born calf, a new-born calf with clinical signs or a clinically healthy but persistently infected calf [1-3]. Experimental primary infections in pregnant cattle during early pregnancy with *N. caninum* (first trimester; e.g. at 70 days of pregnancy) generally produce foetal death and abortion, whereas infection from the second trimester onward (e.g. at 140 days of pregnancy) generally results in clinically healthy but congenitally infected calves [6,7]. Several mechanisms have been proposed to be related to the occurrence of abortion, such as the damage caused by parasite proliferation in the placenta, which jeopardises foetal viability directly by restricting oxygen/nutrition, an immunological imbalance in the placenta, promoting a Th1 response harmful to the foetus, multiplication of parasite in vital organs of the foetus, or the release of prostaglandins that provoke abortion and/or damage to the foetus [1,2,8,9]. The disease outcome is influenced by the maternal immune response in the placenta and the relative immune-competence of the foetus at the time of infection [1,8,9].

In this context, little is known about the influence of the virulence that is inherent to the isolate on transmission and abortion in cattle. Differences in invasion and proliferation capacities, as well as mechanisms of transmigration across biological barriers in vitro, have been described for different isolates and associated with observed variations in pathogenicity and transmission in mice [10,11]. Comparison of tachyzoite-proteome profiles by 2-D DIGE has revealed differences in the expression of proteins that are involved in gliding motility, lytic cycle processes of the parasite, and oxidative stress [12]. Importantly, *N. caninum* isolates exhibit differences in their capacity to cause lesions in cerebral mouse models [13-15], and in their transmission efficacy from dams to offspring [16-18]. However, studies in cattle are limited, and it remains unclear if the differences exhibited by *N. caninum* in vitro and in animal laboratory models could have any repercussions on the outcome of the disease in bovines. An absence of foetal death at day 45 post-infection (pi) in heifers inoculated intravenously at early pregnancy with a low-virulence isolate, Nc-Spain1H, has been reported [19], and differences between two virulent isolates in cattle, Nc-Spain7 and NC-1, were also shown in the timing of foetal death and immunological responses in an early pregnant bovine model [20]. Here, we investigated the pathogenicity of two *N. caninum* isolates with differential virulence, based on their in vitro invasion and proliferation capacities and to their pathogenicity in pregnant mice [10,18,21], in an early pregnant bovine model. Highly virulent Nc-Spain7 and the low-to-moderately virulent Nc-Spain8 were compared to determine their differential influences on transmission, the occurrence of abortion, parasite distribution, parasite burdens and lesions in the foetus, placenta and maternal tissues, and maternal immune responses at systemic level and localised in the placenta.

## Material and methods

### Ethics statement

All protocols involving animals were approved by the Animal Welfare Committee of the Agricultural Research Centre of Mabegondo (CIAM-INGACAL), A Coruña, Spain, following proceedings described in Spanish and EU legislations (Law 32/2007, R.D. 1201/2005 and Council Directive 2010/63/EU). All animals were handled in strict accordance with good clinical practices and all efforts were made to minimise suffering.

### Animals and experimental design

Animals used in this study came from the Holstein Friesian dairy herd of CIAM-INGACAL, which is free of *N. caninum* infection. Thirty-two heifers, aged 20–24 months, were selected after assessing their seronegativity to *N. caninum*, *Leptospira* spp., Infectious Bovine Rhinotracheitis (IBR) virus and Bovine Viral Diarrhoea (BVD) virus by ELISA. These heifers were oestrus synchronised with the administration of: 1) 250 μg of synthetic gonadorelin analogue (Fertagyl, Merck Animal Health, Millsboro, DE, USA) (day 0), 2) 150 μg of synthetic prostaglandin F2α analogue (Dalmazin, Fatro-Ibérica, Barcelona, Spain) (day 7) and 3) 250 μg of Fertagyl (day 9). Twelve hours after the last administration, 2 artificial inseminations, 12 h apart, were carried out using semen from a Friesian *N. caninum*-seronegative bull. Pregnancy was confirmed by ultrasound scanning on day 32 after insemination and 14 pregnant heifers were selected for the experiment. The remaining non-pregnant heifers were oestrus synchronised again with 150 μg of Dalmazin and then mated by natural service with a Friesian bull seronegative for *N. caninum*, *Leptospira* spp., IBR and BVD. Pregnancy was assessed by ultrasound scanning at day 35 after mating, and 3 other pregnant heifers were selected for the study.

Animals pregnant by artificial inseminations (n = 14) were randomly distributed into three experimental groups. Twelve heifers were allocated to groups 1 (G1; n = 6) and 2 (G2; n = 6), which were inoculated intravenously with 10^7^ culture-derived tachyzoites of the Nc-Spain7 and Nc-Spain8 bovine isolates [22], respectively, at 70 days of gestation. Two remaining heifers were allocated to group 3 (G3) and received an inoculated intravenously inoculum of uninfected MARC-145 cells in phosphate-buffered saline (PBS) treated in the same way than tachyzoites at 70 days of gestation. G3 was used as a negative control and a sentinel for adventitious infections until calving (infection-sentinel group). The three pregnant animals mated by natural service were also inoculated with MARC-145 cells in PBS at 70 days of gestation (group 4; G4) and culled at day 39 post-inoculation as a negative control for further analyses (see below). Experimental design is summarised in Table 1.

**Table 1:** Experimental design

**Group**	**Heifer ref.**	**Pregnancy**	**Inocula**	**Animal euthanasia**
**1**	**H2849**	AI	Nc-Spain7	Yes
	**H3237**		10^7^ tachyzoites i.v.	
	**H3255**			
	**H9500**			
	**H9516**			
	**H9522**			
**2**	**H3236**	AI	Nc-Spain8	Yes
	**H3263**		10^7^ tachyzoites i.v.	
	**H9507**			
	**H9510**			
	**H9517**			
	**H9530**			
**3**^**a**^	**H3270**	AI	MARC-145	No
	**H9501**		8 × 10^5^ cells i.v.	
**4**^**b**^	**H3242**	NS	MARC-145	Yes
	**H3247**		8 × 10^5^ cells i.v.	
	**H9518**			

^a^Sentinel-control group (G3): Heifers completed the pregnancy with the birth of two serologically negative calves at 269 and 274 days of pregnancy.

^b^Foetal-control group (G4). Heifers were culled at 39 dpi for further comparative analyses.

*AI*, Artificial insemination; *NS*, Natural service.

i.v., Intravenous inoculation.

### Parasites

Nc-Spain7 and Nc-Spain8 isolates were routinely maintained in a monolayer culture of MARC-145 cells after reactivation from cryovials, as described previously [18]. The experiment was carried out using similar parasite passage numbers in MARC-145 cells following isolation in cell cultures for Nc-Spain7 (passage no.8) and Nc-Spain8 (passage no.13). Inocula were prepared as described previously [18]. Tachyzoites used for inoculations were recovered from MARC-145 cultures when the majority of the parasites were still intracellular. Viable tachyzoite number was determined by Trypan blue exclusion followed by counting in a Neubauer chamber, and the organisms were resuspended in PBS at the required dose of 10^7^ tachyzoites in a final volume of 1 mL. Tachyzoites were administered to heifers within 1 h of harvesting from tissue culture. The viability of tachyzoites was checked in vitro by a plaque assay in MARC-145 cell cultures. Biological characteristics in vitro and in vivo for the Nc-Spain7 and Nc-Spain8 isolates were previously summarised and these isolates categorised as described [21].

### Clinical monitoring and sampling

Cattle were observed daily after inoculations throughout the experimental period. Rectal temperatures were recorded daily from 3 days prior to challenge to 13 days post-infection (dpi) and then, weekly from 14 dpi onward. Animals with temperatures above 39.5 °C were considered to be febrile. Blood samples were collected at day −3 and −1, thereafter twice weekly until 13 dpi, and then once weekly throughout the remainder of the experimental period by jugular veni-puncture in heparinised Vacutainer tubes (Becton Dickinson and Company, Plymouth, UK). Blood samples were immediately processed in order to obtain plasma and peripheral blood mononuclear cells (PBMC) for further analyses.

Transrectal ultrasonography was used to determine foetal viability by monitoring its heart beat. All heifers were monitored once weekly for the three first weeks pi and then twice weekly until detection of foetal death. When ultrasound examinations confirmed foetal death, dams were sedated with xylazine hydrochloride (Rompun; Bayer, Mannheim, Germany) and immediately euthanised by an inoculated intravenously overdose of embutramide and mebezonio iodide (T61; Intervet, Salamanca, Spain). The animals of G4 were euthanised at 39 dpi, i.e. the median time period when foetal deaths were detected in infected groups (G1 and G2), for control of further analyses. The animals of G3 were examined monthly for pregnancy by ultrasound and rectal palpation from the sixth week until calving and calves were kept alive. Post-mortem examination of the heifers was carried out immediately after euthanasia. Foetuses were immediately separated from the placenta. Ten randomly selected placentomes were recovered from each placenta and, when not already separated, the maternal (caruncle) and the foetal (cotyledon) sides of the placenta were immediately and carefully detached by hand. Both the caruncle and cotyledon were transversally cut in slices of 2–3 mm thickness, which were distributed for storage in 10% formalin for histopathological examinations and in RNA*later* (Sigma-Aldrich, Saint Louis, MO, USA) for cytokine mRNA expression analysis and parasite DNA detection by PCR. Foetal tissues included the brain, heart, liver, lungs and a portion of semitendinosus skeletal muscle, which were maintained at −80 °C for DNA extraction and fixation in 10% formalin. Foetal thoracic and abdominal fluids were also collected and maintained at −80 °C for serology and PCR. Heifer tissues including the brain and spinal cord (one half at −80 °C and one half in 10% formalin), masseter skeletal muscle and lymph nodes (retropharyngeal and iliophemoral) were collected for PCR and histopathological analysis.

### Serological analyses: *N. caninum*-specific IgG responses

Specific IgG, IgG1 and IgG2 antibody levels were measured using the CIVTEST Bovis Neospora (Laboratorios HIPRA, Girona, Spain) enzyme-linked immunosorbent assay (ELISA). Data were expressed as a relative index percent (RIPC). A RIPC value ≥ 10 indicates a positive result. The *Neospora*-specific IgG response was measured in foetal fluids by indirect fluorescent antibody test (IFAT) [23]. More details are given in Additional file 1.

### Peripheral blood mononuclear cell (PBMC) isolation, stimulation assays and evaluation of *N. caninum*-specific IFN-γ and IL-4 responses

PBMCs were separated with Histopaque-1077 (Sigma-Aldrich, Madrid, Spain). Cells were cultured in triplicate with: 1) *N. caninum* soluble antigen at 5 μg/mL [23], 2) ConA at 2.5 μg/mL (positive control) and 3) PBS (negative control). After 72 h of culture (37 °C, 5% CO_2_), IFN-γ and IL-4 levels were measured in supernatants by using Bovine IFN-γ and Bovine IL-4 Screening Set kits (Thermo-Fisher Scientific, Rockford, IL, USA). More details are given in Additional file 1.

### Histopathology and immunohistochemical detection of *N. caninum*

After fixation for five days in 10% formalin, maternal brains were coronally cut from frontal cerebrum to medulla, to include 11 different areas, and processed along with the rest of the maternal and foetal tissue samples by standard procedures for haematoxylin and eosin (H&E) staining [24]. Sections from placentome and foetal viscera wax blocks were cut and treated for the immunohistochemical identification of *N. caninum* antigens using an in-house anti-*N. caninum* polyclonal serum according to previously described [24].

### Tissue DNA extraction and PCR determinations

Genomic DNA was extracted from 50–100 mg of maternal and foetal tissue samples and 200 μL of thoracic and abdominal foetal fluids using the Maxwell® 16 Mouse Tail DNA Purification Kit (Promega, Wisconsin, USA). Genomic DNA from placental tissues was simultaneously extracted with total RNA for cytokine expression analysis with TRIzol Reagent (Life Technologies, Pasley, UK). Parasite DNA detection was carried out with nested-ITS1 PCR adapted to a single tube with the external primers TgNN1/TgNN2 and internal primers NP1/NP2 previously described by Hurtado et al. [25] and Buxton et al. [26], respectively. Positive samples by ITS1 PCR from the foetal brain, liver and heart and the placenta were quantified for parasite DNA using real-time PCR [27]. *N. caninum* genotyping was performed on ITS-1 PCR-positive foetal brain samples by the MS5, MS7, MS8 and MS10 markers [28]. More details are given in Additional file 1.

### Quantification of cytokine mRNA expression levels in the placenta

Total RNA for cytokine expression analysis was extracted from placental tissues by a combined method based on the TRIzol Reagent (Life Technologies, Pasley, UK) and Qiagen RNeasy Mini Kit (Qiagen, Hilden, Germany). Reverse transcription was carried out using SuperScript® VILO™ cDNA Synthesis Kit (Invitrogen, Paisley, UK). Primers used for bovine IFN-γ, IL-12p40, TNF-α, IL-4, IL-10 cytokines and the housekeeping gene β-actin were designed using the Primer-3Plus software [29] and are shown in the Additional file 2. Real-time PCR reactions were performed in 20 μL using Power SYBR®PCR Master Mix (Applied Biosystems, Foster City¸ CA, USA), 10 pmol of each primer and 5 μL of diluted cDNA samples in an ABI 7300 Real Time PCR System (Applied Biosystems). The cytokine mRNA level was expressed in fg of cytokine mRNA/mg of tissue. The relative quantification of cytokine mRNA expression levels (*x*-fold change in expression) was carried out by the comparative 2^–ΔΔCt^ method [30]. More details are in Additional file 1.

### Statistical analysis

In order to compare parameters recorded in vivo (rectal temperatures, antibody response and cytokine levels) data from both control groups (G3 and G4) were merged into a unique group. Temperature values and antibody responses for each experimental group were analysed by using the one-way ANOVA test. When statistically significant differences were found, a Tukey’s Multiple Range test was applied to examine all possible pairwise comparisons at each sampling time. Variations in IFN-γ and IL-4 levels, parasite burdens and cytokine mRNA levels were analysed using the non-parametric Mann–Whitney test and Kruskal-Wallis test, followed by a Dunn’s Multiple Range test for all pairwise comparisons. Differences in PCR detection of parasite DNA in foetal tissues were evaluated using the *X*^2^ or Fisher exact F-test. Occurrence of foetal death was analysed by the Kaplan–Meier survival method to estimate the percentage of viable foetuses at each time point [31]. The foetal survival curves of the infected groups were then compared with the Gehan-Wilcoxon test. Statistical significance for all analysis was established with *P <* 0.05. Differences that showed *P* values ≥ 0.05 and < 0.11 were considered to be trending towards statistical signification. All statistical analyses were carried out using SPSS v 15.0 (Chicago, IL, USA) and GraphPad Prism 5 v.5.01 (San Diego, CA, USA) software.

## Results

### Clinical observations

The mean rectal temperatures of infected animals (G1 and G2) increased significantly (> 38.4 °C) between 5 to 7 dpi and thereafter returned to basal levels (Additional file 3). The maximum mean temperature was detected at 5 dpi (*P <* 0.001), when three animals from G1 and two from G2 exhibited fever (> 39.5 °C). No significant difference was found between the infected groups. Rectal temperatures of the uninfected animals (G3 and G4) remained below 38.1 °C throughout the experiment.

Foetal death was detected in all infected heifers between 24 and 49 dpi (Table 2) with median values for foetal survival of 34 dpi in G1 and 41 dpi in G2. Specifically, during the fourth and fifth weeks of infection, foetal death occurred in most of the heifers from G1 (5/6), but only in one G2 animal. The comparative analysis of foetal survival curves between infected groups showed a trend towards statistical significance (*P =* 0.108) with a higher foetal median survival time in G2. Foetal death was not detected in the control groups (G3 and G4). Dams from sentinel-group G3 delivered healthy calves and foetuses from G4 remained alive just prior the euthanasia of their dams on 39 dpi.

**Table 2:** **Detection of foetal death, foetal serology and PCR-detection of *N. caninum *** **in placenta and foetuses**

**Group**	**Foetus ref.**	**Foetal death (dpi)**	**IFAT titre**^**b**^	**Parasite ITS1 PCR detection**^**a**^							
				**Placental tissues**		**Foetal tissues**					
				**Caruncle**	**Cotyledon**	**Brain**	**Liver**	**Heart**	**Lung**	**Muscle**	**Foetal fluids**^**c**^
**1**	**F2849**	34	32	5/5	5/5	4/5	3/3	3/3	3/3	3/3	2/2
**(Nc-Spain7)**	**F3237**	49	128	4/5	5/5	5/5	3/3	0/3	3/3	3/3	2/2
	**F3255**	24	-	5/5	5/5	5/5	3/3	3/3	3/3	2/3	1/2
	F9500 (1)*	34	16	5/5	5/5	5/5	3/3	3/3	3/3	3/3	2/2
	**F9500 (2)**	34	16	4/5	5/5	5/5	3/3	3/3	3/3	3/3	2/2
	**F9522**	34	32	4/5	5/5	5/5	3/3	3/3	3/3	3/3	2/2
	**F9516**	27	-	5/5	4/5	5/5	3/3	3/3	3/3	3/3	2/2
**2**	**F3236**	44	N.D.	3/5	4/5	5/5	2/3	0/3	0/3	2/3	N.D.
**(Nc-Spain8)**	**F3263**	30	-	2/5	5/5	4/5	1/3	2/3	3/3	3/3	2/2
	**F9507**	41	-	4/5	5/5	5/5	3/3	3/3	3/3	3/3	2/2
	**F9510**	41	32	1/5	5/5	5/5	3/3	0/3	3/3	0/3	2/2
	**F9517**	44	64	5/5	5/5	5/5	3/3	3/3	3/3	3/3	2/2
	**F9530**	37	-	5/5	5/5	5/5	2/3	3/3	3/3	2/3	2/2

^a^Fractions represent the number of positive tissue samples/total number of samples tested by nested-ITS1 PCR.

^b^IFAT titres assessed in thoracic fluids.

^c^Parasite detection in one aliquot of thoracic and abdominal foetal fluids.

^*^Heifer H9500 was carrying twins. Both foetuses were independently analysed.

N.D. Not evaluated.

### Pathology and immunohistochemical labelling

#### Gross lesions

At the time of slaughter, all the infected dams showed detachment of more than 75% of the placental surface from the uterus; in most of the cases, the detachment was complete. There was abundant turbid and yellowish liquid in between the separated placenta and the uterus mucosa. The surface of the uterine caruncles was congestive and showed fibrin floccules. One heifer, H9500, from G1 was carrying twins that were considered individual foetuses in further analyses. Foetal viscera were in general oedematous and friable, suggesting autolysis.

#### Microscopic lesions

All the caruncles from infected dams studied, regardless of the group, showed similar lesions in terms of severity and characteristics of the inflammatory infiltrate involved. The caruncles showed a diffuse lesion that affected the entire mid-zone of the studied samples and in some samples it also extended to the arcade and crypt regions of the caruncle. In this area, there was a diffuse mixed inflammatory infiltrate within the septa formed by macrophages, lymphocytes, plasma cells and, less frequently, neutrophils. This infiltrate caused the loss of the normal architecture of the caruncle and the obliteration of the crypts. There were few multifocal to coalescent necrotic areas randomly distributed within the inflammatory infiltrate, where occasional mineralisation of cellular debris was visible.

**Figure 1:**
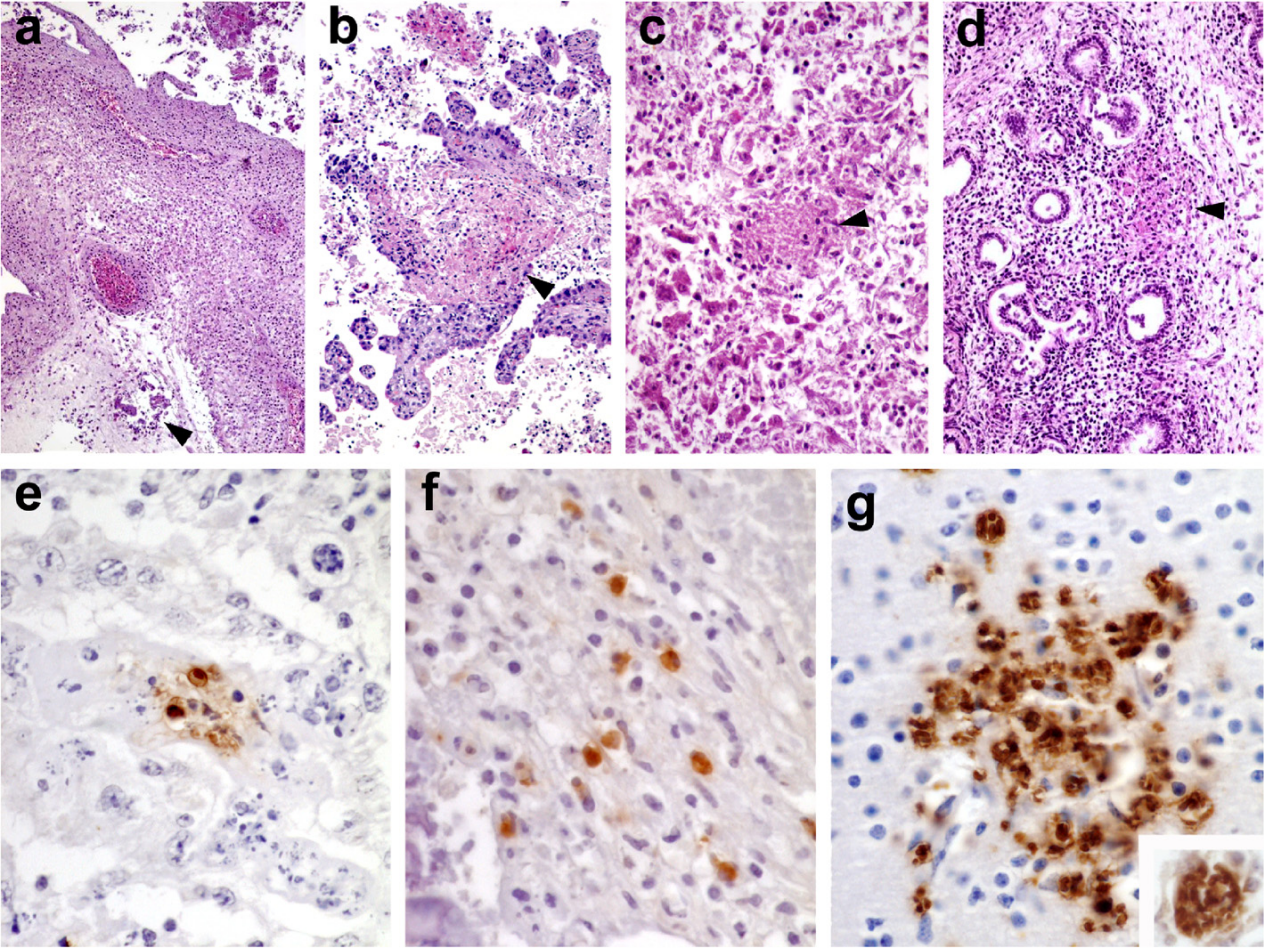
**Histological and immunohistochemical findings in foetal samples. (a)** Cotyledon. Diffuse and dense infiltrate of mononuclear inflammatory cells in the chorioallantoic stroma at the chorionic plate. Note the focus of mineralisation (arrowhead) HE. 15×. **(b)** Cotyledon. Focus of coagulative necrosis in a cotyledonary villus (arrowhead). The necrosis involves throphoblast layers and extends to the mesenchyme underneath. HE. 20×. **(c)** Focus of coagulative necrosis in the liver. HE. 35×. **(d)** Focus of coagulative necrosis in the lung (arrowhead). Note the absence of inflammatory cells. HE. 25×. **(e)** Placentome. Positive immunolabelling (brownish colouration) of parasitophorous vacuoles within cellular debris between foetal and maternal villi. IHC. 140×. **(f)** Placentome. Positive signal (brownish coloration) in the cytoplasm of macrophage-like cells within the inflammatory infiltrate in the caruncular septum. IHC. 140×. **(g)** Brain. Clusters of protozoal organisms immunolabelled (brownish coloration) in the neuroparenchyma with no evident necrosis or related inflammatory infiltration. IHC. 140×. Inset: detail of one of these clusters in which multiple parasite-like structures could be appreciated. IHC. 180 × .

As in the maternal caruncles, all foetal cotyledons studied from infected dams showed similar lesions that were indistinguishable between groups. The most conspicuous lesion in these cotyledons was the presence of a diffuse mononuclear infiltrate formed by lymphocytes and macrophages in the chorioallantoic stroma, especially around big vessels, with occasional areas of mineralisation (Figure 1a). The cotyledons showed extensive autolysis of the trophoblast layer. Sporadically, foci of necrosis in the foetal villi involving the trophoblast layer and the subjacent mesenchyme remained visible (Figure 1b).

Foetal viscera showed extensive autolysis in all cases, impairing the histological evaluation of the samples from two foetuses (one from each group). In the remaining foetuses, the liver showed multifocal necrotic hepatitis. Poorly demarcated necrotic foci appeared randomly distributed in the hepatic parenchyma and there was a striking absence of inflammatory infiltration either in relation to these lesions or in any other area of the liver (Figure 1c). Five foetuses from G2 and one from G1 showed necrotic foci in the lung similar in distribution and characteristics to those described for the liver (Figure 1d). Few glial foci were found in the brain from two foetuses belonging to group G2, while no lesions were found in the samples from group G1.

#### Immunohistochemical findings

*Neospora* antigen was observed in the placenta samples and foetal tissues from all infected dams. The intensity of the labelling was similar among all animals, with no clear difference between groups. Either in placentomes (both caruncular and cotyledonary areas) or foetal samples, positive signal appeared as parasitophorous vacuoles or cytoplasmic labelling (Figure 1f) in cells morphologically compatible with macrophages; this last type of signal was subjectively found more frequently in samples from G1. There was no relationship between the characteristics of the histological lesion and the type of immunohistochemical labelling, although the degree of autolysis in the foetal samples made it difficult to interpret subtle changes in histological features. In the placenta, in addition to these types of labelling, samples from both groups showed positive signal in the form of particulate antigen. Interestingly, in the foetal organs no particulate antigen was found, but there was frequent positive labelling of clusters of organisms (Figure 1g) with no relationship to histological lesions in H&E stained sections. The same number of foetuses from each group (n = 4) showed these clusters in the heart or liver; however, the finding of clusters in the brain was more common in foetuses from group G2 (n = 4) than group G1 (n = 1).

### Parasite distribution and burden in placental and foetal tissues

*N. caninum* DNA was sporadically detected in tissues from two heifers, one from each infected group. Parasite DNA was detected only in one sample from the masseter muscle of one heifer of G1 (H9516, 27 dpi) and in one sample from the brain and the masseter muscle of one heifer of G2 (H9517, 44 dpi).

**Figure 2:**
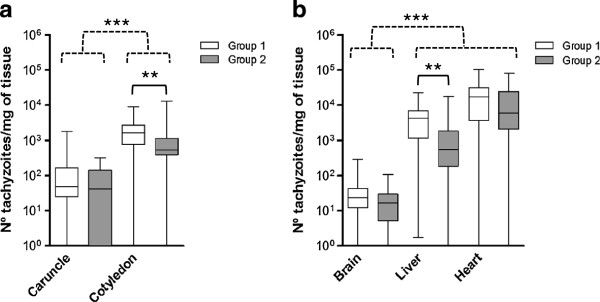
**Median *N. caninum *burdens in placenta and foetal tissues.** Parasite burdens quantified by real-time PCR in placental **(a)** and foetal tissues **(b)** from animals inoculated with 10^7^ Nc-Spain7 tachyzoites (G1) and 10^7^ Nc-Spain8 tachyzoites (G2) (see legend). Box-plot graphs represent the median parasite burden (number of parasites per mg of host tissue), the lower and upper quartiles (boxes) and minimum and maximum values (whiskers). Taking into account that the *N. caninum* detection limit by real-time PCR is 0.1 parasites, all positive samples had > 10^-1^ parasites, and negative samples (0 parasites) were represented on the log scale as < 10^-1^ parasites. (**) over continuous-line brackets indicates significant differences between infected groups in each tissue; *P <* 0.01. (***) over discontinuous-line brackets indicates significant differences between placental and foetal tissues in each infected group; *P <* 0.001.

*Neospora* DNA was detected in placentomes, caruncles and cotyledons, of all infected heifers. With regard to the frequency of detection (Table 2), parasite DNA was observed in 100% of the placentome samples, in 32/35 of caruncles and 34/35 of cotyledon samples (at least in four out of five samples analysed per animal) from G1 and in 20/30 of caruncles and 29/30 of cotyledon samples from G2 (Table 2). The differences in the frequency of parasite detection between the two infected groups was statistically significant for caruncles (*P <* 0.01) but not for cotyledons. The mean parasite burden, measured as the number of tachyzoites per mg of tissue, was significantly higher in cotyledons than in caruncles in both infected groups (Figure 2a). Differences in parasite burden between infected groups were statistically significant in cotyledons (*P* < 0.01) but not in caruncles (*P =* 0.14).

Regarding foetal tissues, the brain, liver, lungs, muscle and foetal fluids from G1 were positive for *N. caninum* DNA for all foetuses (7/7), and the heart was positive in 6/7 foetuses. In G2, parasites were detected in the brain, liver and fluids in all foetuses (6/6), while parasite were found in the lungs and muscle in 5/6 foetuses and the heart in 4/6 foetuses (Table 2). The frequencies of detection were significantly lower in G2 tissues than G1 foetuses (*P* < 0.01). Statistical analysis also demonstrated a higher frequency of detection in the liver (*P* < 0.01) and a trend towards the statistical significance in the muscle and the lung (*P* < 0.09) from G1 compared to G2. The parasite burden, measured as tachyzoites per mg of tissue, was also significantly higher in liver samples from G1 than from G2 (*P* < 0.01), but no differences were found between infected groups in terms of brain and heart parasite burdens (Figure 2b). All samples from G4 were negative.

Brain samples from each infected foetus were also checked by microsatellite analysis, confirming the implication of Nc-Spain7 and Nc-Spain8 isolates in the infection of all animals from G1 and G2, respectively (Additional file 4).

### Specific IgG antibody responses in heifers and foetuses

**Figure 3:**
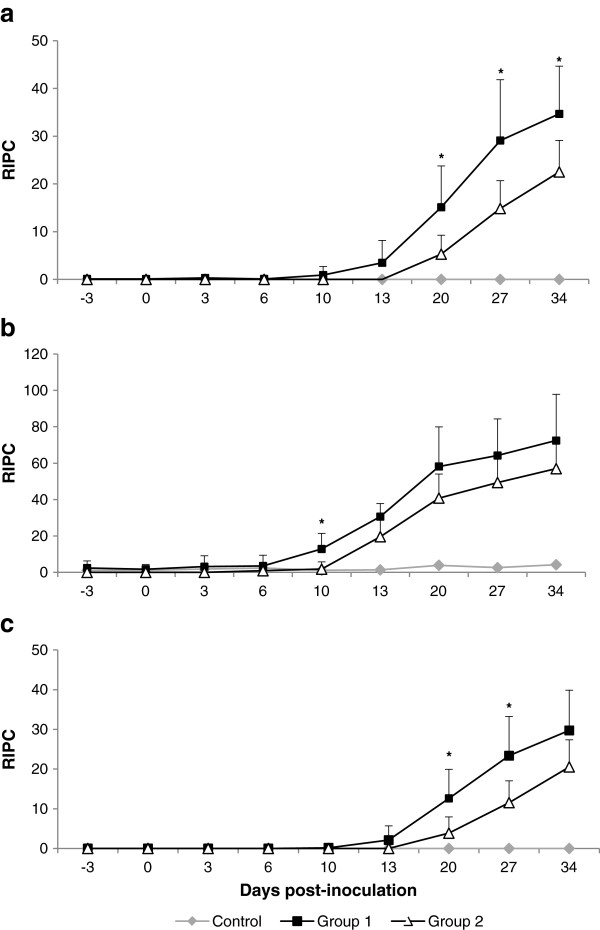
**IgG humoral immune responses.** Serum levels of total IgG **(a)**, IgG1 **(b)** and IgG2 **(c)** antibodies against *N. caninum*. Immunoglobulin levels are expressed as a relative index percent (RIPC) according to formula: RIPC = (OD_405nm_ sample - OD_405nm_ negative control)/(OD_405nm_ positive control - OD_405nm_ negative control) × 100. Each point represents the mean + SD at the different sampling times. (*) Indicates significant differences (*P <* 0.05) between the two infected groups (G1 and G2). Data for 49 dpi are not represented because only one heifer from G1 and two heifers from G2 maintained pregnancy.

*N. caninum* specific antibody responses (total IgG, IgG1 and IgG2) are shown in Figure 3. Total IgG levels (Figure 3a) increased significantly (*P* < 0.001) from 20 dpi in G1 and from 27 dpi in G2 and remained high throughout the study in both infected groups, but there were higher mean values in heifers from G1 than from G2 (*P* < 0.05). When IgG subtypes were determined (Figures 3b and 3c), it was observed that, in both infected groups, the IgG1 response was earlier (from 10 dpi for G1 and 13 dpi for G2) than the IgG2 response (from 20 dpi for G1 and 27 dpi for G2). Significant differences (*P* < 0.05) between infected groups were only found for IgG1 antibody levels at 10 dpi and for IgG2 antibody levels at 20 and 27 dpi, which were higher in G1.

*Neospora*-specific IgG responses were also analysed in foetal fluids by IFAT (Table 2). Seropositive titres were detected in fluids of foetuses that succumbed starting at 34 dpi (104 days of gestation) onwards for G1 and from 41 dpi (111 days of gestation) onwards for G2. Specific IgG responses were never detected in animals and foetuses from control groups (G3 and G4).

### In vitro response of PBMCs: IFN-γ and IL-4 production

**Figure 4:**
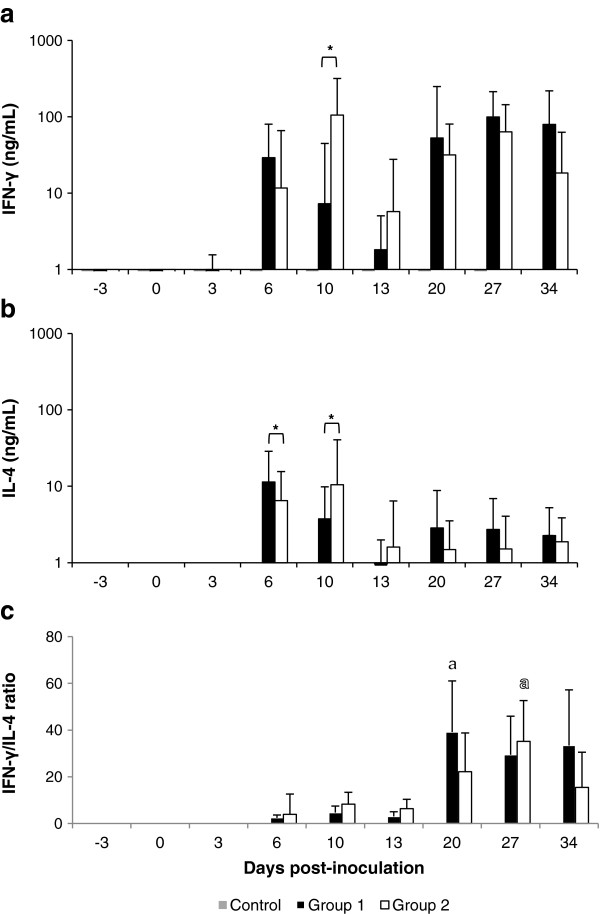
**Peripheral IFN-γ and IL-4 responses.** Concentrations (ng/mL) of IFN-γ **(a)** and IL-4 **(b)** as well as mean IFN-γ/IL-4 ratios **(c)** segregated by *N. caninum* antigen-stimulated PMBCs from heifers inoculated with 10^7^ Nc-Spain7 tachyzoites (G1), 10^7^ Nc-Spain8 tachyzoites (G2) or MARC-145 cells as a control group (G3 and G4) (see legend). Results are presented as median + upper quartile value in **(a)** and **(b)** or as mean + SE in **(c)**. Filled and empty letters “a” indicate the day on which foetal-death was soon after detected in G1 and G2, respectively. (*) Indicates significant differences (*P* < 0.05) between infected groups. Data for 49 dpi are not represented.

The in vitro specific stimulation of PBMCs from heifers of both infected groups triggered a significant (*P* < 0.01) increase of IFN-γ levels from 6 dpi onwards (Figure 4a). However, the time course of IFN-γ production levels differed between cell cultures from G1 and G2, such that levels of production peaked later in the cultures from G1 (27 dpi) than in those from G2 (10 dpi). Regarding the specific IL-4 response (Figure 4b), we also observed that concentrations produced by PBMCs from infected heifers significantly overcame basal levels (pre-infected and/or control animals levels) from 6 dpi onwards (*P* < 0.01). Nevertheless, concentrations of this cytokine were always lower than those of IFN-γ. Moreover, contrary to that observed for IFN-γ production, cultures from G1 were the first to reach the maximum level. Significant differences between infected groups in IFN-γ and IL-4 levels were observed only in the first and second week of infection (Figures 4a and b). None of the control animals (G3 and G4) showed IFN-γ or IL-4 segregation by PBMC assay above basal levels recorded before inoculation throughout the experimental study.

IFN-γ/IL-4 ratios were calculated as an indicator of the balance of the Th1/Th2 cellular immune responses throughout the infection. In the first cultures after infection (6–13 dpi), the IFN-γ/IL-4 ratios were low, with mean values ranging between 2.3 ± 1.2 and 3.3 ± 2.8 in G1 and 5.3 ± 8.7 and 8.0 ± 5.1 in G2. From 20 dpi onwards, the IFN-γ/IL-4 ratios increased significantly in both infected groups, with mean increases about ten times in G1 and five times in G2 (*P* < 0.05). Maximum ratios for these cytokines were reached earlier in G1 than in G2 (20 dpi vs. 27 dpi), and in both groups, this event was concurrent with the first significant increases in IgG2 antibody levels and the appearance of the first foetal deaths.

### Cytokine mRNA expression levels in placental tissues

**Figure 5:**
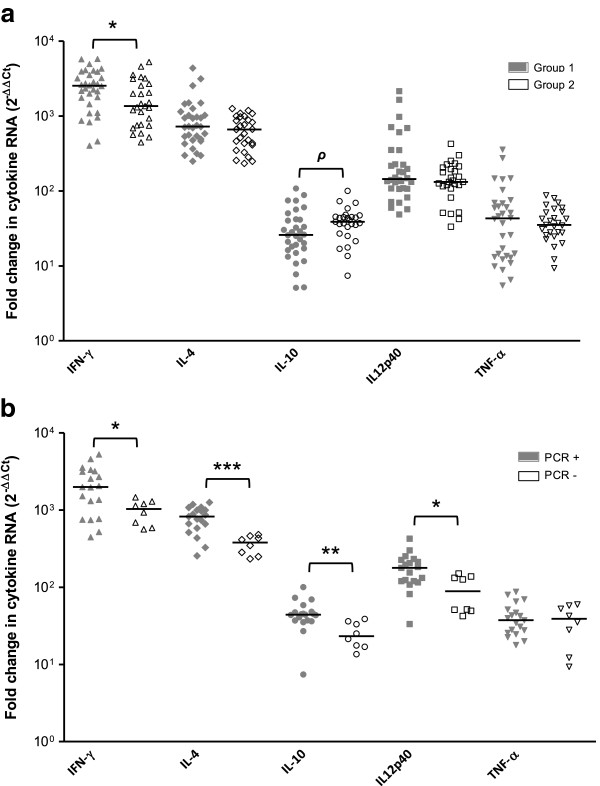
**Cytokine expression profiles in the placenta.** Scatter-plot graphs of relative cytokine expression levels (as *x*-fold change) in infected caruncles from heifers inoculated with 10^7^ Nc-Spain7 tachyzoites (G1), 10^7^ Nc-Spain8 tachyzoites (G2) **(a)** and PCR-positive and PCR-negative caruncles from G2 **(b)**. Horizontal lines represent median values for each group. (*) indicates *P* < 0.05, (**) *P* < 0.01, (***) *P* < 0.001 and (*ρ*) a trend towards significant differences between infected groups **(a)** or between PCR + and PCR- caruncles from G2 **(b)**.

The evaluation of the relative expression levels in placental tissues of infected animals demonstrated a large upregulation of all cytokines. The cytokine fold changes in caruncles and cotyledons of infected animals showed that IFN-γ (1337–2544 fold change, according to the tissues and the infected group) and IL-4 (622–1132 fold) expression were most heavily induced, followed by IL-12p40 (121–258 fold), TNF-α (36–74 fold) and IL-10 (12–39 fold) (Figure 5a). Median fold changes detected in uninfected animals varied from 0.5-2.2 fold for all cytokines, confirming a significant induction of expression for cytokines in infected animals (*P* < 0.0001). The analysis of estimated cytokine mRNA per mg of tissue also revealed significantly higher expression of all cytokines in the maternal side (caruncles) than in the foetal side (cotyledons) (*P* < 0.001) (Additional file 5). Estimated β-actin mRNA levels (housekeeping) in infected caruncles were similar to those assessed in uninfected caruncles (Additional file 5). Nevertheless, the β-actin gene expression in infected cotyledons showed a significant decrease in comparison to uninfected animals, suggesting that RNA integrity could be compromised in these samples.

To investigate the potential role of *N. caninum* isolates in cytokine mRNA expression, we compared the relative levels measured in caruncles from both infected groups (Figure 5a). Interestingly, IFN-γ mRNA expression was significantly more induced in G1 than in G2 (*P* < 0.05). A trend towards significance was also observed when comparing the relative expression of IL-10 between infected groups, with a higher upregulation in animals from G2 (*P =* 0.053). Differences in the expression of IL-4, IL-12p40 and TNF-α were not found. Because differences in the frequency of parasite DNA detection in the caruncles were established between infected groups, we also explored the association between cytokine profile and parasite presence in caruncle sections from G2 by comparing cytokine expression of ITS1 PCR-positive and PCR-negative samples. Significant increases in the expression of IFN-γ, IL-4, IL-10 and IL-12p40 (*P <* 0.05) were found in PCR-positive caruncle sections (Figure 5b).

## Discussion

In the present study, we have compared the clinical outcome, infection dynamics, pathogenic effects in tissues and host immunity induced by two *N. caninum* isolates in cattle after experimental infection at 70 days of pregnancy. Nc-Spain7 and Nc-Spain8 isolates, which were described as high and low-to-moderate virulence isolates, respectively [21], were selected for comparisons as they showed marked differences in virulence as measured by their capacities to produce disease in the offspring delivery of mice infected by *N. caninum* during gestation as well as their proliferation capacities in vitro [10,18]. Furthermore, the high virulence of Nc-Spain7 has recently been confirmed in cattle by comparison studies with the NC-1 isolate in a pregnant bovine model [20]. Experimental infections were conducted by inoculating tachyzoites during early pregnancy because previous studies have shown that the most severe clinical consequence, abortion, occurs with infection during this period [6,7,32]. Foetal death occurs often when cattle are challenged during the first trimester of pregnancy, whereas tachyzoite inoculation during the second and third trimesters of gestation usually results in transplacental transmission of the parasite but not foetal death. *N. caninum* was intravenously administered to facilitate rapid spreading of both isolates, ensuring placental infection before the development of an effective maternal immune response with the potential capacity to limit the infection [33,34]. Inoculation of both isolates resulted in placental and foetal infection, histological lesions and foetal mortality at 3–7 weeks post-inoculation in accordance with previous studies [6,32,33]. Notably, by contrast that observed in pregnant mice, both isolates showed the same clinical outcome. In addition, infection in all heifers was also accompanied by early IFN-γ/IL-4 cytokine production under specific PBMC stimulation, a specific IgG response and an upregulation of cytokine mRNA levels in maternal placenta as described previously [6,17,20,32,33,35,36]. Nevertheless, differences in the timing of foetal death, parasite dissemination in maternal and foetal tissues and the kinetics of peripheral and local immune responses were observed depending on the *N. caninum* isolate used for the infection. These findings may reflect the differences in behaviour displayed by these isolates during in vivo and in vitro in previous studies [10,18].

In accordance with previous reports, a transient rise in body temperature was recorded after infection during the first week with fever peaking at day 5 pi for both groups, likely as a consequence of tachyzoite inoculation and the first cycles of parasite replication in host tissues [20,32,33]. Similar early febrile temperature responses suggest homogeneity in the administrated dose of viable tachyzoites as confirmed for the inocula in vitro.

Foetal death occurred in all heifers, regardless of the isolate, after infection reached the placenta and foetal tissues. Histological lesions in the placenta and foetal tissues were similar to those reported in previous experimental studies of early infection and were characterised by multifocal to coalescent necrotic placentitis [6,19,20,32,33,37]. Bearing in mind that the lesions associated with parasites found in the foetus were scarce, it is tempting to hypothesise that widespread lesions in cotyledons and caruncles denote high tropism of the parasite for the placenta and indeed this might be a key factor for the occurrence of abortion after primary infection during early pregnancy. Previous studies have shown that *N. caninum* was sporadically detected and associated with mild lesions in the placenta or foetuses that survived after subcutaneous inoculation of NC-1 or the intravenous inoculation of the low virulence Nc-Spain1H isolate at 70 days of gestation [19,33]. Nc-Spain1H showed a very low vertical transmission rate (5%) in a pregnant mouse model in comparison with the transplacental transmission of Nc-Spain7 (79%) and Nc-Spain8 (56%) isolates [17,18], suggesting that very limited dissemination to the placenta and crossing to the foetus, as shown for Nc-Spain1H, are required for reduced foetopathy in cattle infected during early pregnancy. Nevertheless, it remains unclear the main cause for foetal mortality after a primary infection during early pregnancy. Tissue damage in the placenta and the foetus caused by direct *N. caninum* multiplication and the actual contribution of the local immune response that develops in the placenta remains to be determined. Histological lesions in the placenta might cause nutrient starvation of the foetus compromising viability. Lesions characteristic for hypoxia were not observed in foetal tissues involved in this study, although the autolysis of the samples impaired the proper evaluation of the foetal samples.

Foetal death was recorded earlier in heifers inoculated with Nc-Spain7 than in those inoculated with Nc-Spain8. Six out of 7 Nc-Spain7-infected foetuses were dead at 5 weeks pi, while 5 out of 6 foetuses infected with Nc-Spain8 remained viable at this time in the pregnancy. Foetal mortality was recorded around 3 weeks pi under similar conditions (10^7^ tachyzoites inoculated intravenously at 70 days of gestation) with the most virulent *N. caninum* isolate Nc-Liverpool [6,32]. Differences in timing of abortion may be due to potential differences in the dynamics of parasite infection for each implicated *N. caninum* isolate and the immune response induced. The isolates used in the current study exhibit in vitro differences in their proliferative capacities [10,21]. This may result in exposure to a higher number of Nc-Spain7 parasites to the placenta and foetus, while Nc-Spain8 may need more time to multiply in the tissues and, therefore, to produce similar lesions and foetal mortality. Furthermore, parasite DNA frequencies of detection and *N. caninum* burden support this hypothesis, as they were significantly greater in placental and foetal tissues infected with Nc-Spain7 than Nc-Spain8. Similar foetal mortality rates to those observed in the present study, 100%, were also recorded when Aberdeen Angus heifers were inoculated intravenously with 10^8^ Nc-Spain7 tachyzoites [20]. Experimental infections with NC-1 parasites revealed 60% foetal mortality at 45 dpi with 10^7^ or 10^8^ tachyzoites as compared with 100% foetal death at 28 dpi when the dose of the inoculum was increased to 5 × 10^8^ tachyzoites [19,20,33]. These findings suggest that the inoculation dose used in this study for both isolates could have attained or overcome the dose threshold necessary for 100% foetal mortality with Nc-Spain7 and Nc-Spain8. Additionally, the route of inoculation or gestational age of infection could influence the abortion rate [33,35]. We could then hypothesise that clearer differences in pathogenicity between Nc-Spain7 and Nc-Spain8 isolates may be demonstrated with a lower tachyzoite dose, alternative inoculation routes (subcutaneous instead of inoculated intravenously) or tachyzoite inoculation during the second or third trimester of pregnancy.

We also examined the peripheral humoral and cell mediated immune responses of pregnant heifers. All infected heifers developed *N. caninum*-specific antibody responses starting from the second week pi with similar dynamics to those observed in previous experimental infections [6,32,33]. Mean maternal antibody responses in Nc-Spain7-infected heifers remained higher than those animals inoculated with Nc-Spain8, possibly due to exposure to more abundant antigen and increased lymphoid stimulation induced by Nc-Spain7 [19,20]. IFN-γ and IL-4 were also produced in antigen-specific PBMC stimulation analyses, at the end of the first and during the second week following infection with *N. caninum* and prior to mounting a specific IgG response. Previous reports have demonstrated that CD4+ T cells from experimentally infected cattle proliferate following stimulation with *N. caninum* antigen and there was upregulation in the expression of IFN-γ and IL-4 mRNAs from 7 to 9 dpi [36]. These findings suggest that soon after infection, the peripheral cellular immune response is not transiently polarised either towards the Th1 or Th2 response. This early cytokine response is likely associated with an innate immune response involving interaction of parasite molecular patterns with recognition receptors (Toll-like receptors) on antigen presenting cells such as dendritic cells [38]. Differences in the kinetics of early IFN-γ/IL-4 responses induced by Nc-Spain7 and Nc-Spain8 isolates were also discerned, with high levels achieved by Nc-Spain7 in the first week. However, the relevance of the early IFN-γ/IL-4 cytokine balance in the control of parasite spreading and prevention of abortion in cattle has yet to be clarified [6]. After innate IFN-γ/IL-4 production, the cell-mediated immune response is clearly skewed towards a predominant Th1 response because a significant increase in the IFN-γ/IL-4 ratio was observed starting at 20 dpi, which coincided with the appearance of the IgG2 response and, soon after, the first cases of foetal death. Previous studies have demonstrated that high levels of IFN-γ in PBMC proliferative analyses are maintained after infection at 70 days of gestation [6,19,20,32,39]. Apparently, a predominant Th1 immune response and high peripheral IFN-γ production is associated with protection against *N. caninum*-induced abortion in natural and experimental infections [34,40-42]. In this study, higher IFN-γ levels were induced by Nc-Spain7 compared to Nc-Spain8 starting at 20 dpi, although no statistical differences were found between both groups. Only significantly higher IFN-γ levels were detected in heifers for the innate response after infection with Nc-Spain8 at 10 dpi. Nevertheless, all dams aborted after the infection and earlier with Nc-Spain7 than with Nc-Spain8. A predominant Th1 response and high levels of peripheral IFN-γ were apparently unsuccessful in preventing the colonisation and multiplication of *N. caninum* in the placenta, maybe due to active parasite proliferation and host dissemination during the first weeks pi in accordance with previous findings [33,37], but later were apparently effective in controlling *N. caninum* systemically.

It is recognised that an efficient immune response against *N. caninum* requires a Th1 cytokine response involving IFN-γ to control parasite multiplication, although an excess of IFN-γ in the placenta may have detrimental effects for gestation and compromise foetal viability [8,9,43]. The Th2 cytokine response triggered at the materno–foetal interface may counteract the effects of inflammatory cytokines to ensure foetal viability, though it may also facilitate parasite proliferation in placental tissues [8,9,43]. In this study, the expression profiles of the studied cytokines in terms of fold change (IFN-γ > IL-4 > IL-12p40 > TNF-α ≥ IL-10) were consistent with those demonstrated in previous studies of early infection when foetal death prevailed [35]. Cytokine mRNA levels showed a strengthened upregulation of IFN-γ, IL-12p40 and TNF-α, which are representatives of the Th1 response, and increased mRNA levels of Th2 (IL-4) and regulatory cytokines (IL-10), confirming a mixed Th1 and Th2 pattern in *N. caninum*-infected caruncles [35,44]. Additionally, a similar Th1/Th2 cytokine ratio is maintained after infection during mid and late gestation but with a minor increase in the expression of Th1 and Th2 cytokines [35,44], which was found to be associated with low frequencies of DNA parasite detection and mild histological alterations in the placenta [6,35]. In this study, we detected higher IFN-γ mRNA levels in caruncles infected with Nc-Spain7 compared to those infected by Nc-Spain8, likely caused by the more rapid multiplication of Nc-Spain7 and thus more rapid antigen production, increasing parasite persistence in caruncles and induction of local responses. Increased levels of IFN-γ in caruncles may have contributed to early foetal mortality recorded for pregnant heifers infected by Nc-Spain7. Interestingly, an increase in mRNA IL-10 levels was instead observed in Nc-Spain8 infected-caruncles, which has been proposed to downregulate IFN-γ expression [44].

Although differences in parasite dissemination between isolates were detected by PCR, no differences were observed between infected groups regarding the severity or characteristics of placental lesions. These findings suggest that, regardless of the isolate, the local immune response developed in the placenta was not able to control the multiplication of the parasite. The fact that parasite burden was much higher in the samples from cotyledons than from caruncles suggests that parasites were actively multiplying on the foetal side of the placenta. Cellular response in cotyledons could not be evaluated in this study due to tissue autolysis and, more than likely, mRNA degradation. Previous studies have suggested that the cytokine upregulation found in the placenta is likely produced by lymphocytes in the maternal side rather than immune cells of foetal origin [35]. The lower burden of parasites found in caruncles suggests that, after caruncle colonisation and formation of tissue lesions, parasite multiplication could be partially controlled by the local maternal response, but it is unable to impede invasion and active, uncontrolled multiplication in the cotyledons. Thus, foetal immunocompetence at the time of infection might play a key role on foetal survival, limiting lesions in foetal tissues [6,35,44]. Maturation of foetal immunity appears to occur gradually for mid gestation [8]. *N. caninum*-specific foetal responses have often been described when infection occurs during the second and third trimesters of gestation onward [39,45,46]. Therefore, when infection occurs during early pregnancy, the foetal immune system is not mature enough to control parasite multiplication, allowing wide spreading through foetal tissues, tissue damage, foetal death and likely caruncle re-infection. Nevertheless, a strong foetal IFN-γ response has been associated with abortion when experimental infection is conducted on day 110 of pregnancy [7]. Interestingly, our results showed that Nc-Spain7-infected foetuses were able to mount a humoral immune response against *N. caninum,* starting at day 104 of pregnancy (day 34 pi), while this response did not appear until the week after (starting at day 111 of pregnancy, day 41 pi) in those infected by Nc-Spain8. These results are in accordance with those described by Bartley et al. [34], who demonstrated a specific humoral immune response at day 98 of gestation (day 28 pi) in one foetus that succumbed after an intravenously inoculation of 5 × 10^8^ NC-1 tachyzoites. The earlier IgG responses detected in Nc-Spain7-infected foetuses compared to Nc-Spain8-infected foetuses may be associated with the earlier and higher stimulation induced by Nc-Spain7 antigens, which is in accordance with their proliferative characteristics [10]. It has been demonstrated that peripheral lymphoid tissues of infected foetuses mature and produce immunoglobulins earlier than those that are uninfected and of the same gestational age [47]. However, the immune response mounted by infected foetuses during early pregnancy was not protective against foetal mortality.

In summary, our results showed variations in the time of foetal death and the differential development of maternal and foetal immune responses based on the biological characteristics of the *N. caninum* isolate implicated in the infection. These observations suggest that new studies are necessary to establish the actual influence of the isolate in the occurrence of abortion in natural infection.

## Competing interests

The authors declare that they have no competing interests.

## Authors’ contributions

JRC conceived the study and participated in its design, drafted the manuscript, participated in inoculation, developed cytokine PCRs, and interpreted PCR data and statistical analysis. DAS participated in inoculation, clinical examination of cattle and necropsy, the development of cytokine PCR, and carried out PCR and RT-PCR analyses. JB, drafted the manuscript, performed the necropsy and sampling of the animals, and carried out histopathological and inmunohistochemical analyses of the samples. MGB, conceived the study and participated in its design, drafted the manuscript and participated in necropsy and sampling of the animals. JACH, participated in PBMC stimulation assays and serological assays. MM, participated in PBMC stimulation assays, clinical examination of animals and held to design the study and draft the manuscript. VP, participated in interpretation of histopathological and immunohistochemical results. LMOM, conceived the study and participated in its design and help in discussion of results and draft the manuscript. MGW, conceived the study and participated in its design, drafted the manuscript, participated in inoculation and clinical examination of animals, carried out PBMC stimulation and serological assays, and interpreted results and statistical analyses. All authors read and approved the final manuscript.

## Supplementary Material

Additional file 1 **Material and methods.** Detailed description of materials and methods sections: 1) Serological analyses, 2) Peripheral blood mononuclear cell (PBMC) isolation, stimulation assays and evaluation of *N. caninum*-specific IFN-γ and IL-4 responses, 3) Tissue DNA extraction and PCR determinations and 4) Quantification of cytokine mRNA expression levels in the placenta: RNA extraction, reverse transcription and real-time PCR. Click here for file

Additional file 2 **Sequences of primers used for cytokine real-time PCR (qPCR) and standard curve data.** Primers used for bovine IFN-γ, IL-12p40, TNF-α, IL-4, IL-10 cytokines and the β-actin were designed using the Primer-3Plus software [29,48] and checked with chromosomal sequences. For all target genes, at least one primer annealed at intron splice junctions or at largely separated exons (for IL-12p40) to prevent amplifications of genomic DNA. Minimal coefficient of regression (R^2^) values for each standard curve, minimal and maximal values for slopes and the maximum and minimum inter-assay coefficient of variation for each PCR target are shown. Click here for file

Additional file 3 **Rectal temperatures.** Mean rectal temperatures (+ SD) of heifers inoculated with 10^7^ Nc-Spain7 tachyzoites (G1), 10^7^ Nc-Spain8 tachyzoites (G2), and MARC-145 cells as control group (G3 and G4) (see legend). Click here for file

Additional file 4 **Multilocus microsatellite genotyping of Nc-Spain7 and Nc-Spain8 isolates in foetal brain samples.** Brain samples from each infected foetus were also checked by microsatellite analysis of the MS5, MS7, MS8 and MS10 markers. *N. caninum* genotyping in foetal samples confirmed the implication of Nc-Spain7 and Nc-Spain8 isolates in the infection of all animals from G1 and G2, respectively. Click here for file

Additional file 5 **Cytokine mRNA levels in the caruncle and cotyledon.** Estimated cytokine mRNA levels in the maternal side (caruncle) and the foetal side (cotyledon) from infected animals (**a**) and estimated β- actin mRNA levels in the maternal side (caruncle) and the foetal side (cotyledon) from infected (G1 and G2) and uninfected animals (G4) (**b**). Box-plot graphs represent the median cytokine expression levels (fg of mRNA per mg of host tissue), the lower and upper quartiles (boxes) and minimum and maximum values (whiskers). (***) indicates significant differences between caruncle and cotyledon from infected animals; *P <* 0.001 (a). Different letters over the boxes, (^a^) and (^b^), denote significant differences in β- actin mRNA levels in pairwise comparisons (b); *P <* 0.001. Click here for file
