# Supplementary material for: Neospora caninum infection during early pregnancy in cattle: how the isolate influences infection dynamics, clinical outcome and peripheral and local immune responses
Source: Vet Res. 2014 Jan 30;45(1):10. doi: 10.1186/1297-9716-45-10 (PMC3922688; doi:10.1186/1297-9716-45-10)
Supplement: Additional file 1 — Material and methods. Detailed description of materials and methods sections: 1) Serological analyses, 2) Peripheral blood mononuclear cell (PBMC) isolation, stimulation assays and evaluation of N. caninum-specific IFN-γ and IL-4 responses, 3) Tissue DNA extraction and PCR determinations and 4) Quantification of cytokine mRNA expression levels in the placenta: RNA extraction, reverse transcription and real-time PCR. [file 1297-9716-45-10-S1.docx]

**Additional file 1 Materials and methods.**

**Serological analyses: *N. caninum*-specific IgG responses**

***Maternal IgG responses***

Blood plasma samples were recovered from heparinised tubes after centrifugation at 1000 × *g* for 10 min and stored at – 80 °C until serological analysis. Specific total IgG antibody levels were measured using the CIVTEST Bovis *Neospora* (Laboratorios HIPRA, Girona, Spain) enzyme-linked immunosorbent assay (ELISA). IgG1 and IgG2 subclasses were also assessed with the CIVTEST Bovis *Neospora* ELISA using peroxidase-conjugated sheep anti-bovine IgG1 and IgG2 antibodies (Serotec, Oxford, UK) at 1:5000 as secondary conjugates. Plasma samples were diluted 1:100 in PBS for testing and analysed in duplicate. Data were expressed as a relative index percent (RIPC), converting the mean value of the optical density (OD_405_) into RIPC according to the following formula: RIPC = (OD_405nm_ sample - OD_405nm_ negative control)/(OD_405nm_ positive control - OD_405nm_ negative control) × 100. A RIPC value ≥ 10 indicates a positive result.

***IgG antibody levels in foetal fluids***

The *Neospora*-specific IgG response was measured in foetal fluids by indirect fluorescent antibody test (IFAT) as previously described [23]. Foetal fluids were diluted at two-fold serial dilutions starting at a 1:8 dilution in PBS to the end point titer. Unbroken tachyzoite membrane fluorescence at titre ≥ 16 was considered a positive reaction.

**Peripheral blood mononuclear cell (PBMC) isolation, stimulation assays and evaluation of *N. caninum*-specific IFN-γ and IL-4 responses**

Buffy coat obtained after blood centrifugation was collected and diluted 1:1 in sterile PBS at room temperature. The cell suspension was layered over Histopaque-1077 (Sigma-Aldrich, Madrid, Spain) and centrifuged at 400 × *g* for 45 min. PBMC were collected from the interface, washed three times in PBS and resuspended in RPMI 1640 medium (Sigma-Aldrich, Madrid, Spain) supplemented with 10% foetal bovine serum, 100 µg/mL streptomycin sulphate and 100 U/mL penicillin (Sigma-Aldrich, Madrid, Spain). Cells, adjusted to a concentration of 10^6^/mL, were distributed in 24-well tissue culture plates (Nunc, Roskilde, Denmark) and cultured in triplicate with: 1) *N. caninum* soluble antigen at 5 µg/mL [23], 2) ConA at 2.5 µg/mL (positive control) and 3) PBS (negative control). After 72 h of culture at 37 °C in a humidified atmosphere with 5% CO_2_, plates were centrifuged and cell-free supernatants were collected and stored at -80 °C until analysis. IFN-γ and IL-4 levels in supernatants were measured by using Bovine IFN-γ and Bovine IL-4 Screening Set kits following the manufacturer’s recommendations (Thermo-Fisher Scientific, Rockford, IL, USA). The cytokine concentrations were calculated from a standard curve generated with recombinant cytokines provided with the kits.

**Tissue DNA extraction and PCR determinations**

***DNA extraction***

Genomic DNA was extracted from 50 - 100 mg of maternal and foetal tissue samples and 200 µL of foetal fluids using the commercial kit Maxwell^®^ 16 Mouse Tail DNA Purification Kit, developed for the automated Maxwell^®^ 16 System (Promega, Wisconsin, USA) following the manufacturer’s recommendations. Genomic DNA from placental tissues was simultaneously extracted with total RNA for cytokine expression analysis with TRIzol Reagent (Life Technologies, Pasley, UK) from the inter-phase and phenol-chloroform phase after removing the aqueous phase containing total RNA, adding 500 µL of a solution containing 4M guanidine isothiocyanate, 0.05M sodium citrate and 1M Tris-base, and precipitating with isopropanol. The concentration of DNA for all samples was determined by spectrophotometry and adjusted to 50-100 ng/µL.

***Neospora DNA detection by nested-ITS1 PCR***

Parasite DNA detection was carried out with nested-ITS1 PCR adapted to a single tube. The single-tube nested-ITS1 external primers TgNN1/TgNN2 and internal primers NP1/NP2 were previously described by Hurtado et al. [25] and Buxton et al. [26], respectively. Each reaction was performed in a final volume of 25 μL under PCR conditions described by Hurtado et al. [25]. ITS1 PCR was carried out with three samples of each of 11 different areas cut coronally from the maternal brain and spinal cord. Three samples from the masseter muscle and the lymph nodes from each heifer were also examined by ITS1 PCR. From each foetus, five samples from the brain and three from the heart, the lungs, the liver and the semitendinosus muscle and one aliquot of thoracic and abdominal fluids were tested by ITS1 PCR. Five placentomes (five maternal caruncles and five foetal cotyledons) from each animal were analysed by ITS1 PCR as well. Negative controls, including reactions without a template and DNA samples from the foetal-control group (G4) were included in each round of DNA extraction and PCR. In each batch of amplifications, positive PCR controls with a quantity of *N. caninum* genomic DNA equivalent to 10 and 1 tachyzoite were also included according to the sensitivity of the ITS1 PCR (DNA equivalent to 1 tachyzoite). Ten µL aliquots of the PCR products were visualised under UV light in a 1.5% agarose/ethidium bromide gel to detect *N. caninum*-specific amplification.

***Neospora-DNA quantification by real time PCR (qPCR)***

Positive samples detected by nested-PCR from the foetal brain, liver and heart and the placental tissues were quantified for parasite DNA using real-time PCR (qPCR). We used primer pairs from the *N. caninum* Nc-5 sequence to quantify parasites and primers from the 28S rRNA gene to quantify bovine DNA under amplification conditions previously described [26]. Reactions were performed in a final volume of 20 μL using Power SYBR^®^PCR Master Mix (Applied Biosystems, Foster City¸ CA, USA), 20 pmol of each primer and 200 ng of DNA in a ABI 7300 Real Time PCR System (Applied Biosystems). All DNA samples were analysed in duplicate. The *N. caninum* number was calculated by the interpolation of average Ct values on two standard curves: 1) equivalent to 5 × 10^5^ to 5 × 10^-1^ tachyzoites with 10-fold serial dilutions in a solution of bovine genomic DNA and 2) a curve of 400 ng, 200 ng, 100 ng, 40 ng, 20 ng, 4 ng, and 1 ng of genomic DNA for bovine DNA quantifications. Parasite number in tissue samples (parasite burden) was expressed as parasite number/mg bovine tissue.

***Multiplex multilocus nested-PCR microsatellite genotyping in foetal tissues***

*N. caninum* genotyping was performed on ITS-1 PCR-positive foetal brain samples using a multiplex multilocus nested-PCR for the amplification of the MS5, MS7, MS8 and MS10 markers as previously described [28]. Microsatellite allele assignment was performed according to the sizes determined by capillary electrophoresis and sequencing of the MS10 and MS7 markers using a 48-capillary 3730 DNA analyser (Applied Biosystems) and Big Dye Terminator v3.1 Cycle Sequencing Kit (Applied Biosystems) at the Unidad Genómica del Parque Científico de Madrid.

**Quantification of cytokine mRNA expression levels in the placenta: RNA extraction, reverse transcription and real-time PCR (qPCR)**

***RNA extraction***

Total RNA for cytokine expression analysis was extracted from placental tissues (caruncles and cotyledons) by means of a combined method based on the TRIzol Reagent (Life Technologies, Pasley, UK) and Qiagen RNeasy Mini Kit (Qiagen, Hilden, Germany) procedures. Briefly, a total of 100 mg of tissue (caruncle or cotyledon) was homogenised using a rotor–stator homogeniser Polytron PT-MR 1600R (Kinematica Ag, Littau, Switzerland) into 1 mL of TRIreagent following the manufacturer’s recommendations until collection of the aqueous phase containing total RNA. Then, RNA purification was completed by Qiagen RNeasy Mini Kit following the manufacturer’s specifications. RNA samples were processed by on-column DNase digestion using 4U of Ambion® DNase I (RNase-free) during purification. The concentration of RNA for all samples was determined by spectrophotometry.

***Reverse transcription***

Reverse transcription was carried out in a 20 μL reaction using 2.5 μg of total RNA and the master mix SuperScript® VILO™ cDNA Synthesis Kit (Invitrogen, Paisley, UK) which includes reverse transcriptase enzyme (SuperScript® III-RT), random primers and a recombinant ribonuclease inhibitor for cDNA synthesis. Caruncle and cotyledon cDNA reactions diluted 1:25 and 1:10, respectively, were used in qPCRs to analyse cytokine mRNA expression.

***Real-time PCR (qPCR)***

Primers used for bovine IFN-γ, IL-12p40, TNF-α, IL-4, IL-10 cytokines and the housekeeping gene β-actin are shown in Additional file 2. Primers were designed using the Primer-3Plus software [29] and checked with chromosomal sequences using the BioEdit Sequence Alignment Editor v.7.0.1 (Copyright_ 1997–2004 Tom Hall, Ibis Therapeutics, Carlsbad, CA, USA). For all target genes (cytokines and β-actin), at least one primer annealed at intron splice junctions or at largely separated exons (for IL-12p40) to prevent amplifications of genomic DNA. Real-time PCR reactions were performed in a final volume of 20 μL using Power SYBR^®^PCR Master Mix (Applied Biosystems, Foster City¸ CA, USA), 10 pmol of each primer and 5 µL of diluted cDNA samples in an ABI 7300 Real Time PCR System (Applied Biosystems) with the following amplification conditions: 95 °C for 10 min followed by 40 cycles at 95 °C for 15 s and 60 °C for 1 min. For each target gene, a six-point standard curve was included in each batch of amplifications based on 10-fold serial dilutions starting at 10 ng/µL of plasmid DNA in which the full-length cDNA containing the gene fragments used as templates in qPCR were cloned. All DNA samples and standard curves were analysed in duplicate and the average Ct was used to determine mRNA expression for each sample. The cytokine mRNA expression level was calculated by the interpolation of the average Ct in plasmid standard curves and then adjusted to the number of fg per ng of total RNA/cDNA equivalent and expressed in fg of cytokine mRNA/mg of tissue. The relative quantification of cytokine mRNA expression levels (*x*-fold change in expression) was carried out by the comparative 2^–ΔΔCt^ method as previously described [30]. The ΔCt for each sample and cytokine was calculated by subtracting the Ct value for the β-actin gene (normaliser) from the Ct values for each cytokine for each sample. Then, the ΔΔCt was calculated by subtracting the mean ΔCt values for G4 as baseline samples (mean Ct for each cytokine – mean β-actin Ct from all uninfected caruncles and cotyledons) from the ΔCt values determined for all *N. caninum* infected samples. Relative fold increases or decreases were then assessed as 2^–ΔΔCt^.
