# Supplementary material for: Neospora caninum infection during early pregnancy in cattle: how the isolate influences infection dynamics, clinical outcome and peripheral and local immune responses
Source: Vet Res. 2014 Jan 30;45(1):10. doi: 10.1186/1297-9716-45-10 (PMC3922688; doi:10.1186/1297-9716-45-10)
Supplement: Additional file 2 — Sequences of primers used for cytokine real-time PCR (qPCR) and standard curve data. Primers used for bovine IFN-γ, IL-12p40, TNF-α, IL-4, IL-10 cytokines and the β-actin were designed using the Primer-3Plus software [29,48] and checked with chromosomal sequences. For all target genes, at least one primer annealed at intron splice junctions or at largely separated exons (for IL-12p40) to prevent amplifications of genomic DNA. Minimal coefficient of regression (R2) values for each standard curve, minimal and maximal values for slopes and the maximum and minimum inter-assay coefficient of variation for each PCR target are shown. [file 1297-9716-45-10-S2.docx]

**Additional File 2:** Sequences of primers used for cytokine real-time PCR (qPCR) and standard curve data.

| **Target^a^** | **Primer** | **Primer sequences (5’-3’)** | **Product size (bp)** | ***R*^2 b^** | **Slope^c^** | **CV (%)^d^** |
| --- | --- | --- | --- | --- | --- | --- |
| IFN-γ (NM_174086.1) | QIFN-UP* | 5’-GATTCAAATTCCGGTGGATG-3’ | 110 | 0.9937 | (-3.47) – (-3.30) | 2.44_(-8)_ - 1.08_(-7)_ |
|  | QIFN-RP | 5’-TTCTCTTCCGCTTTCTGAGG-3’ |  |  |  |  |
| IL-4 (M77120.1) | QIL4-UP* | 5’-CTGCCCCAAAGAACACAACT-3’ | 169 | 0.9946 | (-3.33) – (-3.54) | 1.89_(-6)_ - 0.55_(-7)_ |
|  | QIL4-RP | 5’-GTGCTCGTCTTGGCTTCATT-3’ |  |  |  |  |
| IL-10 (NM_174088.1) | QIL10-UP*^,1^ | 5’-TGCTGGATGACTTTAAGGGTTACC-3’ | 60 | 0.9985 | (-3.27) – (-3.42) | 4.76_(-4)_ - 2.43_(-6)_ |
|  | QIL10-RP | 5’-AAAACTGGATCATTTCCGACAAG-3’ |  |  |  |  |
| IL-12p40 (NM_174356.1) | QIL12-UP | 5’-AGTACACAGTGGAGTGTCAG-3’ | 157 | 0.9963 | (-3.10) – (-3.30) | 2.14_(-6)_ – 0.90_(-3)_ |
|  | QIL12-RP | 5’-TTCTTGGGTGGGTCTGGTTT-3’ |  |  |  |  |
| TNF-α (EU276079.1) | QTNF-UP* | 5’-CCAGAGGGAAGAGCAGTCC-3’ | 126 | 0.9972 | (-3.37) – (-3.45) | 1.88_(-8)_ – 0.23_(-7)_ |
|  | QTNF-RP*^,1^ | 5’-GGAGAGTTGATGTCGGCTAC-3’ |  |  |  |  |
| β- actin (NM_173979.3) | BACTIN-UP* | 5’-ACACCGCAACCAGTTCGCCAT-3’ | 216 | 0.9961 | (-3.62) - (-3.74) | 2.54_(-5)_-1.70_(-3)_ |
|  | BACT216-RP | 5’-GTCAGGATGCCTCTCTTGCT-3’ |  |  |  |  |

^a^ NCBI accession numbers are for bovine cDNA sequences used in primer design. Primer annealing was also checked with the *Bos taurus* genomic DNA sequences of chromosome 5 for IFN-γ, chromosome 7 for IL-4 and IL12p40, chromosome 16 for IL-10, chromosome 23 for TNF-α and chromosome 25 for β-actin in the NCBI database (http://www.ncbi.nlm.nih.gov/nuccore).

^b^ Coefficient of regression of standard curves based on 10-fold dilutions (10^-3^ – 10^-8^) of 10 ng/µL from plasmid stocks. Ct values increased linearly until the level of 10^-8^ dilution of all plasmids. Minimal *R*^2^ values for each PCR target in all amplification batches.

^c^ Standard curve slopes. Minimal and maximal values for slopes for each PCR target in all amplification batches.

^d^ Inter-assay coefficient of variation. CV values indicate the maximum and minimum CVs of all points from standard curves for each PCR target run in this study. Number subscript indicates curve point for CV values.

* Indicates primers annealing at intron splice junctions. No amplification products were detected when bovine genomic RNA free-DNA samples were tested with cytokine primers (data not shown).

^1^ Primers previously described by Rosbottom et al [35].
