# Supplementary figures and images for: Neospora caninum infection during early pregnancy in cattle: how the isolate influences infection dynamics, clinical outcome and peripheral and local immune responses
Source: Vet Res. 2014 Jan 30;45(1):10. doi: 10.1186/1297-9716-45-10 (PMC3922688; doi:10.1186/1297-9716-45-10)

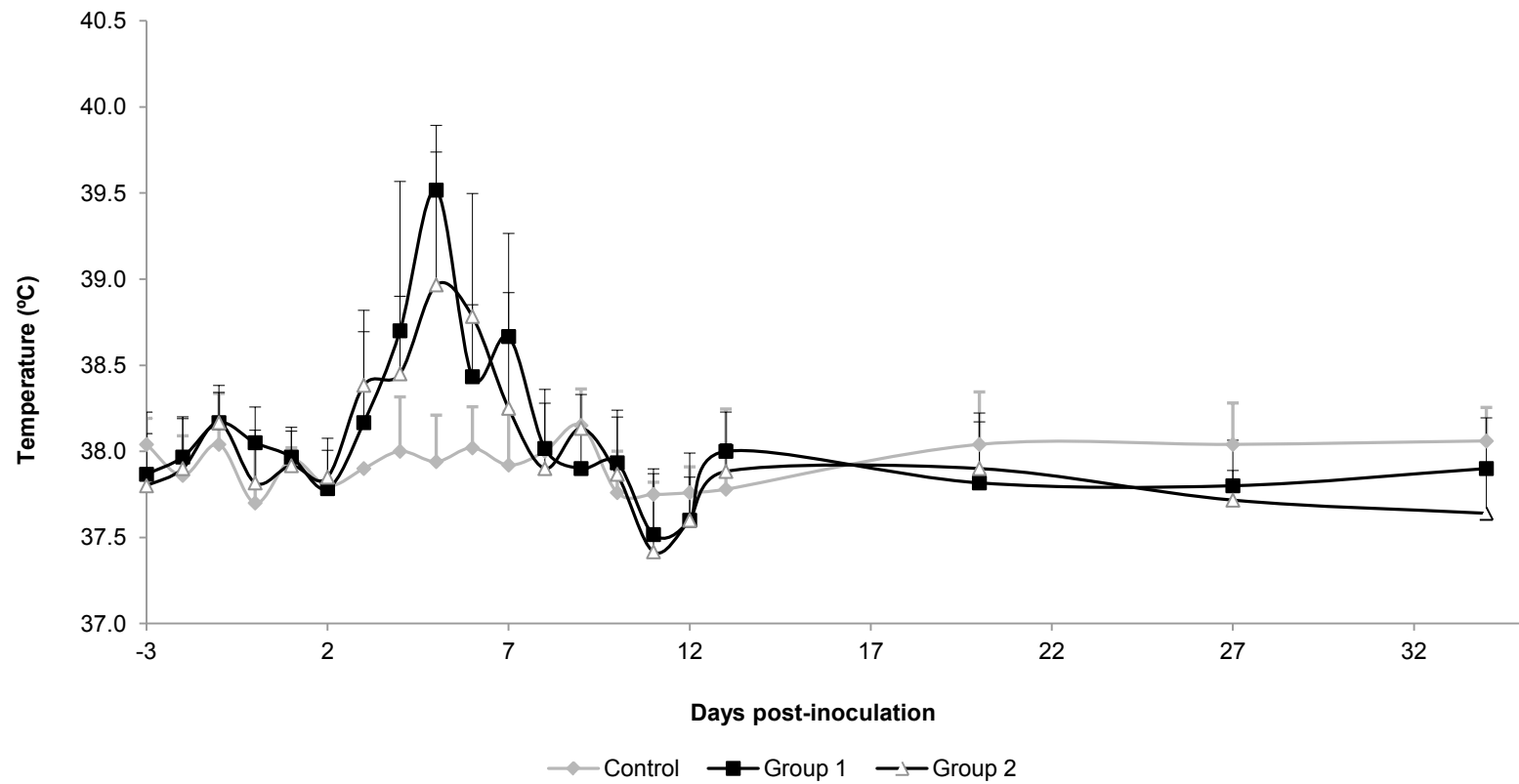

Supplement: Additional file 3 — Rectal temperatures. Mean rectal temperatures (+ SD) of heifers inoculated with 107 Nc-Spain7 tachyzoites (G1), 107 Nc-Spain8 tachyzoites (G2), and MARC-145 cells as control group (G3 and G4) (see legend). [file 1297-9716-45-10-S3.pdf]

**a**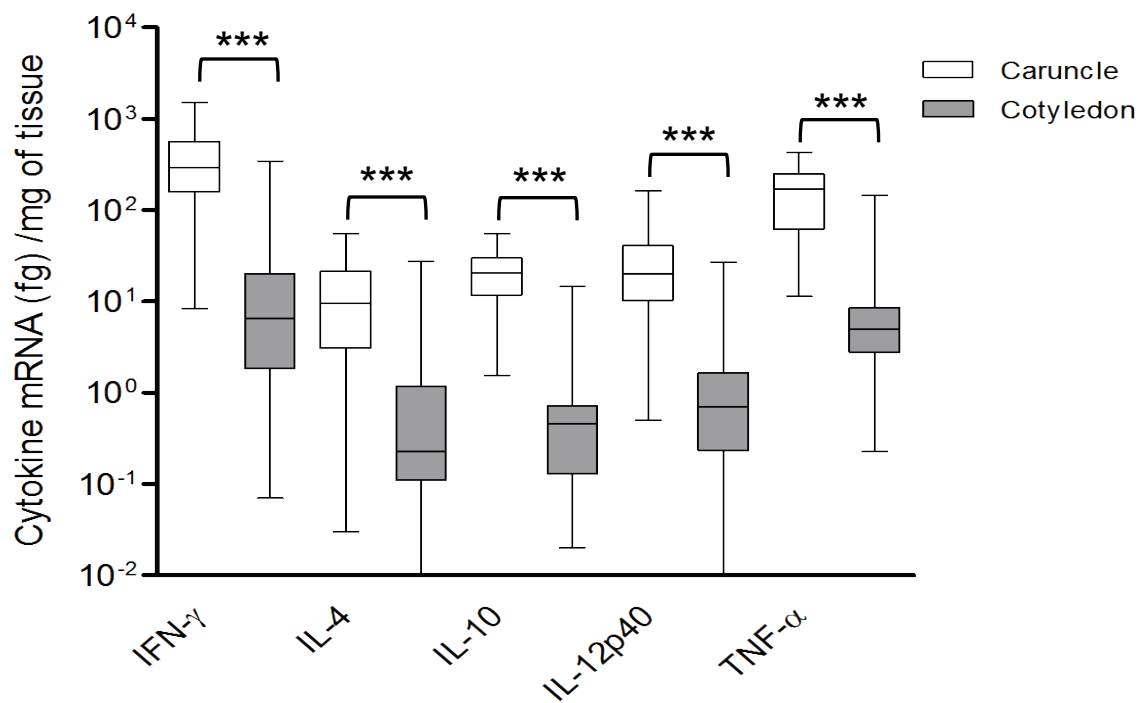**b**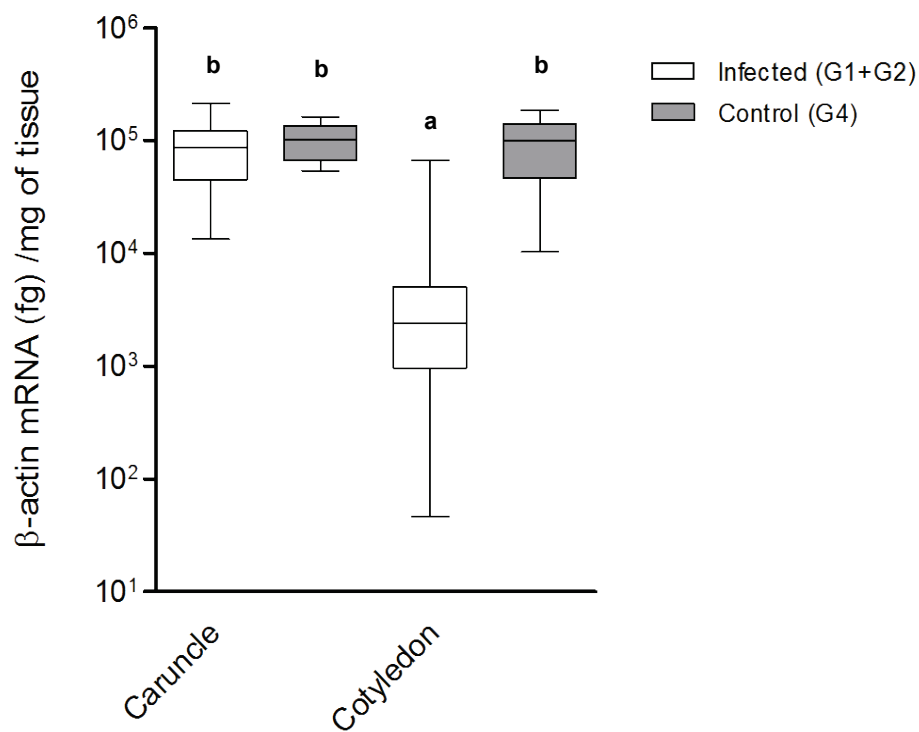

Supplement: Additional file 5 — Cytokine mRNA levels in the caruncle and cotyledon. Estimated cytokine mRNA levels in the maternal side (caruncle) and the foetal side (cotyledon) from infected animals (a) and estimated β- actin mRNA levels in the maternal side (caruncle) and the foetal side (cotyledon) from infected (G1 and G2) and uninfected animals (G4) (b). Box-plot graphs represent the median cytokine expression levels (fg of mRNA per mg of host tissue), the lower and upper quartiles (boxes) and minimum and maximum values (whiskers). (***) indicates significant differences between caruncle and cotyledon from infected animals; P < 0.001 (a). Different letters over the boxes, (a) and (b), denote significant differences in β- actin mRNA levels in pairwise comparisons (b); P < 0.001. [file 1297-9716-45-10-S5.pdf]
