# Supplementary material for: Neospora caninum infection during early pregnancy in cattle: how the isolate influences infection dynamics, clinical outcome and peripheral and local immune responses
Source: Vet Res. 2014 Jan 30;45(1):10. doi: 10.1186/1297-9716-45-10 (PMC3922688; doi:10.1186/1297-9716-45-10)
Supplement: Additional file 4 — Multilocus microsatellite genotyping of Nc-Spain7 and Nc-Spain8 isolates in foetal brain samples. Brain samples from each infected foetus were also checked by microsatellite analysis of the MS5, MS7, MS8 and MS10 markers. N. caninum genotyping in foetal samples confirmed the implication of Nc-Spain7 and Nc-Spain8 isolates in the infection of all animals from G1 and G2, respectively. [file 1297-9716-45-10-S4.docx]

**Additional file 4 Multilocus microsatellite genotyping of Nc-Spain7 and Nc-Spain8 isolates in foetal brain samples.**

| **Group** | **Foetus Ref.** | **Microsatellite profile** | | | |
| --- | --- | --- | --- | --- | --- |
|  |  | MS5 | MS7 | MS8 | MS10 |
|  |  | CG-(*TA*)_n_-TGTA-GG^a^ | ATAA-(*TA*)_n_ | AC-(*AT*)_n_-GG | (*ACT*)_7_-(*AGA*)_n_-(*TGA*)_9_ |
| **1** | **F2849** | 10* | 10 | 16 | 6.23.10 |
|  | **F3237** | NA | NA | 16 | 6.23.10 |
|  | **F3255** | 10 | 10 | 16 | 6.23.10 |
|  | **F9500 (1)*** | 10 | 10 | 16 | 6.23.10 |
|  | **F9500 (2)** | 10 | 10 | 16 | 6.23.10 |
|  | **F9522** | 10 | 10 | 16 | 6.23.10 |
|  | **F9516** | 10 | 10 | 16 | 6.23.10 |
| **2** | **F3236** | 15 | 18 | 15 | 6.18.10 |
|  | **F3263** | 15 | 18 | 15 | 6.18.10 |
|  | **F9507** | 15 | 18 | NA | 6.18.10 |
|  | **F9510** | 15 | 18 | NA | 6.18.10 |
|  | **F9517** | NA | 18 | 15 | 6.18.10 |
|  | **F9530** | 15 | 18 | 15 | 6.18.10 |
| **Nc-Spain7** | | 10 | 10 | 16 | 6.23.10 |
| **Nc-Spain8** | | 15 | 18 | 15 | 6.18.10 |

^a^ Microsatellite marker and microsatellite sequence.

^*^ Allele number is assigned by the number of repeats (n) in the repetitive sequence (indicated in italics) as described [28].

NA: not amplified, not assigned.
